# Examining prevalence and predictors of pulmonary hypertension in adults with idiopathic pulmonary fibrosis: a population-based analysis in the United States

**DOI:** 10.25122/jml-2023-0324

**Published:** 2024-01

**Authors:** Rupak Desai, Nishanth Katukuri, Sai Gautham Kanagala, Nitin Ghadge, Alpha James, Borzoo Tajdin, Akhila Nalla, Sai Diksha Vutukuru, Devi Meghana Kotharu, Avinash Kadiyala, Ankit Vyas, Priyadarshi Prajjwal, Ikechukwu Ogbu

**Affiliations:** 1 Independent Outcomes Researcher, Atlanta, GA, USA; 2 Department of Internal Medicine, Mayo Clinic, Rochester, MN, USA; 3 Department of Internal Medicine, New York Medical College, New York City Health Hospitals Metropolitan, NY, USA; 4 Independent Researcher, Albany, NY, USA; 5 Department of Medicine, Bukovinian State Medical University, Chernivtsi, Ukraine; 6 Independent Researcher, Windsor, Ontario, Canada; 7 Department of Internal Medicine, MNR Medical College, Telangana, India; 8 Department of Medicine, Deccan College of Medical Sciences, Hyderabad, Telangana, India; 9 Department of Internal Medicine, Baptist Hospitals of Southeast Texas Beaumont, TX, USA; 10 Department of Internal Medicine, Bharati Vidyapeeth University Medical College, Pune, India; 11 Department of Internal Medicine, Mountainview Hospital Sunrise GME, Las Vegas, NV, USA

**Keywords:** pulmonary hypertension, pulmonary fibrosis, prevalence, predictors/risk factors, mortality, outcomes, cost

## Abstract

Pulmonary hypertension (PH) often complicates idiopathic pulmonary fibrosis (IPF), a progressive parenchymal lung disease. We investigated predictors of PH in IPF hospitalizations in the United States. We identified IPF hospital- izations with or without PH using the National Inpatient Sample (2018) and relevant ICD-10-CM codes. We com- pared demographics, comorbidities, PH prevalence, and its multivariable predictors adjusted for confounders among patients with IPF. In 2018, 30,335 patients from 30,259,863 hospitalizations had IPF, of which 8,075 (26.6%) had PH. Black (41%), Hispanic (21.3%), and female (28.7%) patients had higher rates of PH compared to white patients (25%). The IPF-PH cohort was hospitalized more often in urban teaching (77.7% vs. 72.2%), Midwest, and West hospitals vs. non-PH cohort. Comorbidities including congestive heart failure (2.08 [1.81–2.39]), valvular disease (2.12 [1.74–2.58]), rheumatoid arthritis/collagen vascular disease (1.32 [1.08–1.61]) predicted higher odds of PH. The PH-IPF cohort was less often routinely discharged (35.4%) and more likely to be transferred to intermediate care facilities (22.6%) and home health care (27.1%) (*P* < 0.001). The PH-IPF group had higher rates of all-cause mortality (12.3% vs. 9.4%), cardiogenic shock (2.4% vs. 1%), dysrhythmia (37.6% vs. 29%), and cardiac arrest (2.7% vs. 1.5%) vs. non-PH cohort (all *P* < 0.001). Patients with PH-IPF also had longer hospital stays (9 vs. 8) and a higher median cost ($23,054 vs. $19,627, *P* < 0.001). Nearly 25% of IPF hospitalizations with PH were linked to higher mortality, extended stays, and costs, emphasizing the need to integrate demographic and comorbidity predictors into risk stratification for improved outcomes in patients with IPF-PH.

## INTRODUCTION

Idiopathic pulmonary fibrosis (IPF) is a spontaneous interstitial lung disease of unknown etiology that often results in progressive loss of lung function through thickening and scarring of the lung tissue. The precise etiology of IPF is still unclear despite consid- erable research, hence the term 'idiopathic'. The typical survival time following diagnosis for this illness is 2 to 5 years, and it is linked to severe morbidity and death [1]. A complex interplay of genetic, environmental, and host factors has been proposed. IPF has been associated with *TERT, MUC5B*, and *SP-C* gene mutations [2,3]. Smoking, air pollution, and occupational and environmental risks have been shown to contribute to IPF [4]. Inflammation, fibrosis, and epithelial damage complicate IPF pathophysiology. Collagen-producing myofibroblastic foci cause scarring of lung tissue [1]. IPF manifests in cough, dyspnea, fa- tigue, weight loss, and finger clubbing, while pulmonary function tests reveal restrictive ventilatory abnormalities and reduced dif- fusing capacity [4]. Auscultation may detect bibasilar crackles.

Secondary pulmonary hypertension (PH) develops as a result of known underlying risk factors, the most prevalent of which are pulmonary and cardiac conditions, one of which is IPF. The in- creased pulmonary arterial pressure in IPF arises from a pre-ex- isting condition or situation rather than being a primary disorder of the pulmonary vasculature. Although not completely under- stood, the pathogenesis of PH in patients with IPF is thought to be multifactorial, involving hypoxic vasoconstriction, aug- mented inflammation, and fibrosis of the pulmonary vasculature [5]. A secondary PH develops in 30–50% of patients with IPF [5], worsening many healthcare outcomes, including mortality. Patients with IPF who also experience PH have a much poorer survival rate [5]. In the early stages, or when initially diagnosed, PH affects 10% of patients with IPF [6], further increasing as IPF progresses, with 32% of patients with advanced IPF awaiting lung transplants [7]. More recent studies suggested even more alarming rates of 32–50% [8]. IPF significantly affects quality of life and healthcare utilization and costs. Patients with IPF need regular medical consultations, diagnostic tests, and medical man- agement, which can be costly and time-consuming. A consider- able amount of Medicare resources was allocated to patients with IPF, costing the US healthcare system about $2 billion annually, medication excluded [9].

Despite their significance, the incidence of PH and the associ- ated risk factors in US patients with IPF have not been extensive- ly studied. This study examined the incidence and risk factors for PH in patients with IPF using a large US database of hospitaliza- tions to help bridge this knowledge gap. ICD-10-CM codes were used to assess the frequency of PH in hospitalized patients with IPF and identify demographic and clinical risk factors.

## MATERIAL AND METHODS

The study population consisted of individuals hospitalized in the United States with a diagnosis of IPF in 2018. The inclusion cri- teria for the study were age ≥18 years and a diagnosis of IPF, which was identified using ICD-10-CM code J84.112. The study sample was selected from the National Inpatient Sample (NIS) – a publicly accessible US dataset that is part of the Healthcare Cost and Utilization Project (HCUP), funded by the Agency for Healthcare Research and Quality (AHRQ). NIS data con- sist of approximately 35 million annual in-hospital admissions from more than 1,000 non-federal acute care institutions in 45 states, excluding long-term acute care and rehabilitation facili- ties (https://hcup-us.ahrq.gov/nisoverview.jsp). Considering that NIS is a de-identified dataset, no Institutional Review Board ap- proval was required or sought.

### Primary outcome and variables

The primary outcome of the study was to determine the preva- lence and independent risk factors for non-group 1 or secondary PH, as identified using ICD-10-CM code I27.2, among hospital- ized individuals with IPF. The prevalence of PH was calculated as the percentage of patients with IPF and a PH diagnosis out of the total number of patients with IPF included in the study. Logistic regression analysis was applied to identify predictors of PH in patients with IPF. The study also analyzed the impact of demographic and clinical variables on the risk of developing PH in patients with IPF.

Other variables that were analyzed as potential predictors of PH in patients with IPF included age, gender, race, length of stay, and comorbidities such as chronic obstructive pulmonary disease (COPD), hypertension, diabetes mellitus, obesity, hyper- lipidemia, heart failure, liver disease, collagen disorders, smoking, alcohol abuse, anemia, depression, history of cancer, and prior history of stroke and myocardial infarction. Logistic regression was performed to identify the factors that significantly increased the risk of PH in patients with IPF.

### Statistical analysis

**Figure 1:**
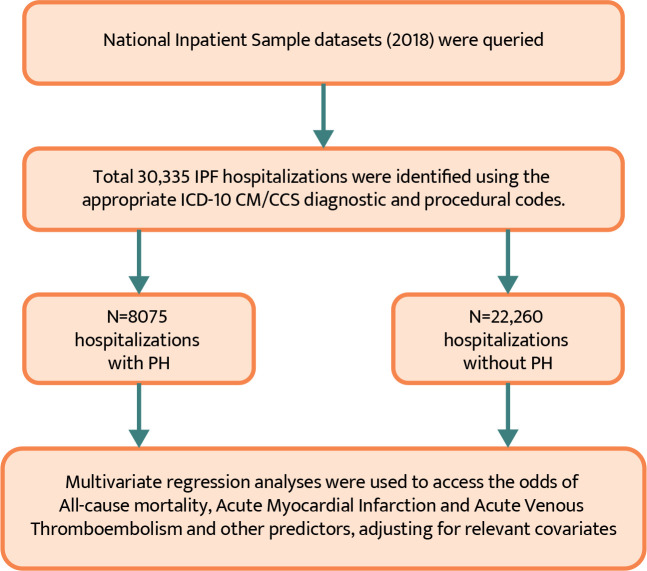
Flow diagram illustrating the patient selection process

The analysis was performed using SPSS software (v25.0, IBM Corp., Armonk, NY, USA). A univariate binary logistic regres- sion was used to determine the relationship between each po- tential predictor variable and the outcome variable (presence or absence of PH). The potential predictors included in the regres- sion were age, sex, race, payer type, hospital region, hospital bed size, hospital teaching status, hospital location, admission type, length of stay, and comorbidities, including COPD, heart failure, diabetes mellitus, and others. A multivariate logistic regression analysis was used to identify the independent predictors of PH in patients with IPF (Figure 1). The adjusted odds ratios (ORs) and respective 95% confidence intervals (CIs) were calculated to esti- mate the strength and direction of the association between each predictor variable and the outcome variable. All statistical tests were two-sided, and a *P* value of less than 0.05 was considered statistically significant.

## RESULTS

In 2018, a total of 30,259,863 hospitalizations were reported in the US, of which 30,335 had a diagnosis of IPF and 8,075 of PH. Among all hospitalizations with IPF, 8,075 (26.6%) were comorbid with PH (Table 1). Female patients with IPF had a greater prevalence of PH than male patients (28.7% vs. 25.1%, *P* < 0.001), while black patients had a higher frequency of the disease than white (41% vs. 25%, *P* < 0.001) and Hispanic pa- tients (24.6%, *P* < 0.001), respectively. Compared to the non-PH IPF cohort, more patients with PH-IPF were admitted to urban teaching hospitals (77.7% vs. 72.7%, *P* < 0.001), and hospitals lo- cated in the Midwest (25.9% vs. 22.9%, *P* < 0.001) and the West (22.1% vs. 18.9%, *P* < 0.001). The complete clinical and demo- graphic characteristics of the study population can be found in Supplementary Table 1.

**Table 1:** Demographic characteristics of hospitalized patients with Idiopathic Pulmonary Fibrosis, with and without Secondary Pulmonary Hypertension

	With Secondary Pulmonary Hypertension		Without Secondary Pulmonary Hypertension		Total
	*n*	%	*n*	%	*n*
Male	4,325	25.1	12,940	74.9	17,265
Female	3,750	28.7	9,320	71.3	13,070
White	5,550	25.0	16,610	75	22,160
Black	960	41.7	1,340	58.3	2,300
Hispanics	805	24.6	2,470	75.4	3,275
Asian or Pacific Islanders	175	21.3	645	78.7	820
Native Americans	85	36.2	150	63.8	235
Others	255	30.4	585	69.6	840
**Total**	**8,075**	**100**	**22,260**	**100**	**30,335**

Furthermore, compared to patients with IPF without PH, the PH-IPF cohort had significantly higher odds of being comorbid with congestive heart failure (aOR 2.08, 95% CI, 1.81–2.39), val- vular disease (aOR 2.12, 95% CI, 1.74–2.58), rheumatoid arthri- tis or collagen vascular disease (aOR 1.32, 95% CI, 1.08–1.61), but not AIDS (aOR 2.15, 95% CI, 0.59–7.79), obesity (aOR 1.14, 95% CI, 0.94–1.37), renal failure (aOR 1.06, 95% CI, 0.91–1.25), or being a smoker (aOR 1.11, 95% CI, 0.87–1.42). The PH-IPF cohort had significantly lower odds of hypertension (aOR 0.67, 95% CI, 0.58–0.76) (Table 2).

**Table 2:** Adjusted^*^ odds ratios of carrying selected comorbidities and risk factors for patients hospitalized with idiopathic pulmonary fibrosis with vs. without pulmonary hypertension

Comorbidity/Risk Factor	aOR	95% CI	*P* value
Congestive heart failure	2.08	[1.81–2.39]	<0.001
Valvular heart disease	2.12	[1.74–2.58]	<0.001
Pulmonary circulation disease	3.00	[2.23–3.97]	<0.001
Rheumatoid arthritis/collagen vascular disease	1.32	[1.08–1.61]	0.006
Hypertension	0.67	[0.58–0.76]	<0.001
Alcohol abuse	0.97	[0.60–1.57]	0.915
Drug abuse	0.53	[0.29–0.99]	0.046
AIDS	2.15	[0.59–7.79]	0.245
Obesity	1.14	[0.94–1.37]	0.185
Renal failure	1.06	[0.91–1.25]	0.447
Smoking	1.14	[1.00–1.30]	0.059
Diabetes mellitus	1.02	[0.88–1.17]	0.824
Hypothyroidism	1.04	[0.89–1.21]	0.630
Liver disease	0.70	[0.49–1.01]	0.058

aOR, Adjusted odds ratio; CI, confidence interval; *P* < 0.05 was considered statistically significant. ^*^Multivariable regression analyses adjusted for clinical and demographic characteristics of the study population are found in Supplementary Table 1.

Supplementary Material

Regarding patient discharge, the PH-IPF cohort was less like- ly to be routinely discharged (35.4% vs. 41.7%) and more likely to be transferred to home health care (27.1% vs. 22.9%, *P* < 0.001 for all). The PH-IPF group had higher all-cause mortality (12.3% vs. 9.4%, *P* = 0.048), experienced more often cardiogenic shock (2.4% vs. 1%), dysrhythmia (37.6% vs. 29%), and cardiac arrest (2.7% vs. 1.5%) compared to the non-PH IPF group (all *P* values <0.001) (Table 3) (Supplementary Table 1). However, acute myocardial infarction (aOR 0.82, 95% CI, 0.65–1.03) and acute venous thromboembolism (aOR 1.10, 95% CI, 0.88–1.36) were not significant compared to the non-PH IPF group (Table 3). Furthermore, the PH-IPF group had a longer length of stay (9 days vs. 8 days) and was charged a higher median cost ($23,054 vs. $19,627, *P* < 0.001 for all; Table 4).

**Table 3:** Adjusted^*^ odds ratios of selected outcomes among patients with idiopathic pulmonary fibrosis with vs. without pulmonary hypertension

Outcome	aOR	95% CI	*P* value
All-cause mortality	1.22	[1.00–1.49]	<0.001
Acute Myocardial Infarction	0.82	[0.65–1.03]	0.085
Acute Venous Thromboembolism	1.10	[0.88–1.36]	0.396

aOR, Adjusted odds ratio; CI, confidence interval; *P* < 0.05 was considered statistically significant. ^*^Multivariable regression analyses adjusted for clinical and demographic characteristics of the study population are found in Supplementary Table 1.

**Table 4:** Comparison of continuous outcomes between IPF patients with and without pulmonary hypertension

Outcome	With PH		Without PH		Median	*P* value
Length of stay (days)	4	(3–8)	5	(3–9)	4	<0.001
Total charges (USD)	10515	(6,344–19,627)	11891	(7,239–23,054)	10515	<0.001

Numbers in parentheses are interquartile range (IQR) of respective medians; PH, pulmonary hypertension; *P* < 0.05 was considered statistically significant

## DISCUSSION

IPF is a progressive, debilitating lung disease characterized by a buildup of scar tissue in the lungs, leading to reduced lung func- tion and impaired gas exchange. PH is a common complication in patients with IPF, characterized by high blood pressure in the pulmonary arteries, which can lead to right-sided heart failure and decreased quality of life. PH is associated with worse out- comes in patients with IPF, including increased morbidity and mortality, as well as increased healthcare utilization and costs. Early detection and management of PH in patients with IPF is crucial for improving clinical outcomes and quality of life.

The current study highlights several predictors of PH in pa- tients with IPF. These findings can help identify high-risk patients with IPF who may benefit from early PH screening and manage- ment. The study found that 26.6% of hospitalized patients with IPF had a diagnosis of PH, with a higher prevalence in female patients compared to male patients. Similar results were seen in a study published in 2012 [10]. The study recruited 126 patients with IPF and found a high prevalence of PH (39.7%, 50/126), defined by echocardiography as pulmonary artery pressure (PAP) greater than 36 mmHg. A higher prevalence of PH was observed among smokers and female patients. Another study aimed to determine the prevalence of PH in patients with IPF and other interstitial lung diseases using transthoracic echocardiography [11]. A total of 239 subjects were enrolled. The prevalence of PH increased from 28.9% to 46.0% when a mean pulmonary arterial pressure (mPAP) of >20 mmHg was used to define PH. While the prevalence was higher in female patients, no statisti- cally significant differences were observed using either definition. The current study supports the higher prevalence of PH among female patients with IPF. However, a study of USA Medicare beneficiaries of ≥ 65 age observed IPF rates higher than previ- ously stated (incidence of 93.7 cases per 100,000 persons/years and prevalence rates of 202.2 and 494.5 cases per 100,000 in 2001 and 2011, respectively) [12]. Other studies suggested an in- creasing prevalence and a stable or increasing incidence of IPF elsewhere. Most concluded higher prevalence and incidence rates among men and with increasing age, especially after 75 years.

Among patients with IPF, black patients had the highest fre- quency of PH (41.7%). This corroborates previous studies that have shown higher rates of PH among black individuals. Collard *et al*. [13] observed that African American patients with IPF were more likely to have PH compared to Caucasian ones. Another study found that non-Hispanic black and Hispanic patients with IPF and PH had lower survival rates following lung transplants compared to non-Hispanic white patients [14]. These disparities remained significant despite adjustment for transplantation sta- tus, medical comorbidities, and socioeconomic status and may have resulted from a worse lung function at presentation. Ge- netic, environmental, and social health determinants may also explain racial differences. For example, black individuals have a higher prevalence of hypertension and other cardiovascular disease risk factors [14]. These data show that black patients with IPF may require focused PH screening and management. Healthcare providers should be aware of this racial disparity and consider race-specific risk factors when assessing and managing PH in patients with IPF.

The current study also found higher odds of congestive heart failure, valvular disease, rheumatoid arthritis/collagen vascular disease, and lower odds of hypertension, liver disease, alcohol abuse, and drug abuse in the PH-IPF cohort. These findings sug- gest that the presence of specific comorbidities can increase the risk of developing PH in patients with IPF. These comorbidities may have important clinical implications as they can affect disease prognosis and treatment outcomes. For example, congestive heart failure and valvular disease are associated with increased morbid- ity and mortality in patients with IPF and PH [15]. Other comor- bidities include obstructive sleep apnea, pulmonary thromboem- bolism, and systemic hypertension [16,17]. These comorbidities may contribute to the development of PH in patients with IPF and may worsen the prognosis and quality of life.

The current study found that patients with PH-IPF had worse discharge outcomes compared to those with non-PH IPF. Spe- cifically, patients with PH-IPF were less likely to be routinely dis- charged and more likely to be transferred to intermediate care fa- cilities and home health care. The PH-IPF group also had higher rates of all-cause mortality, cardiogenic shock, dysrhythmia, and cardiac arrest compared to the non-PH IPF group. These find- ings suggest that PH is a prognostic factor in IPF, as patients with PH-IPF have worse clinical outcomes and utilize higher healthcare costs.

This study confirms prior findings that PH is a predictor of IPF mortality, significantly worsening IPF prognosis. Pulmonary arterial hypertension (PAH) is common in advanced IPF and has been shown to severely reduce survival [7]. Right-heart catheter- ization revealed PAH in 31.6% of patients with IPF, marked by a lower diffusing capacity for carbon monoxide (DLCO) and in- creased oxygen requirement. PAH detection could help with dis- ease progression, prioritization of transplantation, and manage- ment. In a separate study, patients with IPF and PH underwent a series of clinical evaluations [18], including echocardiography, the 6-Minute Walk Test (6MWT), and pulse oximetry (SpO2). Echocardiography proved to be more effective in detecting IPF-associated PAH compared to other tests, except for right car- diac catheterization. Another study of 101 patients with IPF who underwent right heart catheterization to assess the effect of pul- monary artery pressure on survival found that higher mPAP and lower forced vital capacity (FVC) at the time of initial assessment were independent prognostic factors [19]. Respiratory failure was the leading cause of mortality for 60 of the 101 participants in the study. Another study demonstrated that higher pulmonary artery-to-aorta (PA: A) ratios can predict higher mPAP in patients with IPF at initial examination [20]. IPF patients with a PA: A > 0.9 had a worse prognosis. These findings emphasize the need for early detection and treatment of PH in patients with IPF to improve clinical outcomes, hospitalization rates, healthcare costs, and quality of life.

### Limitations

The current investigation utilized the extensive national database, the National Inpatient Sample (NIS), to examine the predictors of PH among hospitalized individuals with IPF in the United States. Although the study offers valuable insights into predictors of PH in patients with IPF, it is important to acknowledge a few limitations when interpreting the results. Firstly, the retrospective design and reliance on administrative data pose limitations. Despite the NIS database being the largest publicly available source of inpatient data in the US, its reliability is affected by the quality of docu- mentation in medical records, which can vary among hospitals and healthcare providers. Secondly, the absence of long-term fol- low-up data restricts understanding of changes in PH prevalence and predictors among adults with IPF over time. Additionally, the lack of specific laboratory data, such as pulmonary function tests and biomarkers, hinders a comprehensive understanding of the relationship between biochemical markers and PH prevalence in the studied population. Lastly, the study is restricted by the unavail- ability of medication information, which prevents the examination of the effects of specific drugs on the prevalence and progression of PH in individuals with IPF.

Furthermore, considering the retrospective design of the cur- rent study, a cause-and-effect relationship between the predictors and the development of PH in patients with IPF cannot be deter- mined. Additionally, only hospitalized patients with IPF were in- cluded in this study, which renders these findings inapplicable to patients with IPF managed in outpatient settings. Moreover, the study only included data from 2018, and as such, the results may not reflect the current prevalence and predictors of PH among patients with IPF. Despite these limitations, our study provides valuable insights into the prevalence and predictors of PH in hos- pitalized patients with IPF in the United States and highlights the need for further research to better understand the impact of PH on clinical outcomes in this patient population.

### Future directions

Hospitalized patients with IPF in the US and worldwide may have different rates of PH. Future research could investigate this further. To understand the worldwide disease burden, comparing the incidence and prevalence of PH in hospitalized patients with IPF in different countries may be useful. The genetic and molec- ular causes of PH in patients with IPF could also be investigated. These pathways could lead to specific PH therapies for patients with IPF, improving outcomes and quality of life. The present study showed that patients with IPF and PH had a longer dura- tion of stay and a higher healthcare cost utilization than those without PH. Future research should examine how PH affects healthcare expenditures and resource consumption. The present study showed multiple predictors of PH in patients with IPF, in- cluding advanced age, female gender, COPD, and heart failure.

## CONCLUSION

This retrospective analysis of the 2018 NIS data examined the prevalence and independent risk factors of PH in hospitalized patients with IPF. The prevalence of PH was 26.6% in hospital- ized patients with IPF. Advanced age, female gender, COPD, and heart failure predicted the presence of PH in patients with IPF. This study emphasizes the need for diagnosing and treating PH in patients with IPF who carry known risk factors. The study also indicates that black patients are at a higher risk for developing PH compared to other racial groups. Race and ethnicity should be considered when treating and researching PH in IPF. Clini- cians may need to monitor patients with IPF for the development of PH, as the latter worsens clinical outcomes and healthcare expenditures.
