# Supplementary material for: Examining prevalence and predictors of pulmonary hypertension in adults with idiopathic pulmonary fibrosis: a population-based analysis in the United States
Source: J Med Life. 2024 Jan;17(1):35–40. doi: 10.25122/jml-2023-0324 (PMC11080510; doi:10.25122/jml-2023-0324)
Supplement: Supplementary file 1 [file JMedLife-17-035-s001.pdf]

Supplementary Material

Supplementary Table 1. Demographic and clinical characteristics of the study population

Variable	With Secondary Pulmonary Hypertension		Without Secondary Pulmonary Hypertension		Total	*P* value
	n	%	n	%	n	
**Age at admission (years)**						<0.001
18-44	275	1.2	160	2	43	
45-64	3,850	17.3	1,520	18.8	5,370	
≥65	18,135	81.5	6,395	79.2	24,530	
Sex						<0.001
Male	12,940	58.1	4,325	53.4	17,265	
Female	9,320	41.9	3,750	46.4	13,070	
**Race**						<0.001
White	16,610	76.2	5,550	70.9	22,160	
Black	1,340	6.1	960	12.3	2,300	
Hispanic	2,470	11.3	805	10.3	3,275	
Asian or Pacific Islander	645	3	175	2.2	820	
Native American	150	0.7	85	1.1	235	
Others	585	2.7	255	3.3	840	
**Median household income national quartile for patient ZIP Code**						<0.001
0-25th	4,855	22.1	1,950	24.6	6,805	
26-50th	5,915	26.9	2,030	25.6	7,945	
51-75th	5,610	25.6	2,020	25.5	7,630	
76-100th	5,570	25.4	1,935	24.4	7,505	
**Primary expected payer**						0.015
Medicare	17,495	78.7	6,355	78.8	23,850	
Medicaid	1,055	4.7	455	5.6	1,510	
Private including HMO	2,975	13.4	1,005	12.5	3,980	
Self-pay	270	1.2	95	1.2	365	
No charges	25	0.1	11		36	
Others	415	1.9	140	1.7	555	
**Location/teaching status of hospital**						<0.001
Rural	1,690	7.6	525	6.5	2,215	
Urban non-teaching	4,505	20.2	1,275	15.8	5,780	
Urban teaching	16,065	72.2	6,275	77.7	22,340	
**Region of hospital**						<0.001
Northeast	4,310	19.4	1,360	16.8	5,670	
Midwest	5,095	22.9	2,090	25.9	7,185	
South	8,650	38.9	2,840	35.2	11,490	
West	4,205	18.9	1,785	22.1	5,990	
**Comorbidities**						
Hypertension (complicated)	13,465	60.5	4,665	57.8	18,130	<0.001
Diabetes Mellitus	7,345	33	2,700	33.4	10,045	0.472

© 2024 by the authors. This article is an open access article distributed under the terms and conditions of the Creative Commons Attribution (CC BY 4.0) license.

Supplementary Material

Supplementary Table 1. Continued. Demographic and clinical characteristics of the study population

Variable	With Secondary Pulmonary Hypertension		Without Secondary Pulmonary Hypertension		Total	*P* value
	n	%	n	%	n	
Hyperlipidemia	11,100	49.9	3,940	48.8	15,040	0.099
Smoking	10,225	45.9	3,815	47.2	14,040	0.043
Obesity	2,815	2.6	1,190	14.7	4,005	<0.001
Peripheral vascular disease	1,590	7.1	710	8.8	2,300	<0.001
Renal Failure	4,340	19.5	1,870	23.2	6210	<0.001
Chronic pulmonary disease	9,165	41.2	3,745	46.4	12,910	<0.001
Congestive Heart Failure	5,045	22.7	3,215	39.8	8,260	<0.001
Valvular Heart Disease	1,660	7.5	1,250	15.5	2,910	<0.001
Liver Disease	1,005	4.5	295	3.7	1,300	0.001
Lymphoma	195	0.9	35	0.4	230	<0.001
Personal History of Cancer	2,785	12.5	915	11.3	3,700	0.006
Metastatic cancer	475	2.1	115	1.4	590	<0.001
Solid tumor without metastasis	680	3.1	210	2.6	890	0.038
Rheumatoid Arthritis or collagen vascular diseases	1,935	8.7	1015	12.6	2,950	<0.001
Coagulopathy	1,735	7.8	710	8.8	2,445	0.005
Deficiency Anemias	4,555	20.5	2,040	25.3	6,595	<0.001
Alcohol abuse	425	1.9	135	1.7	560	0.175
Drug abuse	335	1.6	70	0.9	405	<0.001
Psychoses	580	2.6	160	2	740	0.002
Other Neurological disorders	1,910	8.6	5,050	6.3	6,960	<0.001
Depression	3,000	13.5	1,030	12.8	4,030	0.102
Prior myocardial infarction	2,025	9.1	655	8.1	2,680	0.008
Prior PCI	215	1	70	0.9	285	0.43
Prior CABG	1,875	8.4	735	9.1	2,610	0.062
Prior transient ischemic attack/stroke	1,675	7.5	515	6.4	2,190	0.001
Prior VTE	1,680	7.5	680	8.4	2,360	0.012
Cardiogenic shock	195	2.4	230	1	425	<0.001
Dysrhythmia	3,040	37.6	6,465	29	9,505	<0.001
Cardiac arrest including VF	220	2.7	340	1.5	560	<0.001
**Disposition of patient**						<0.001
Routine	9,285	41.7	2,855	35.4	12,140	
Transfer to short-term hospitals	825	3.7	205	2.5	1,030	
Other transfers SNF, ICF	4,890	22	1,825	22.6	6,715	
Home healthcare	5,090	22.9	2,185	27.1	7,275	
Total	22,260	100	8,075	100	30,335	

PCI, Percutaneous intervention; CABG, Coronary artery bypass grafting; VTE, Venous thromboembolism; HMO, Health maintenance organization; SNF, Skilled nursing facility; ICF, Intermediate care facility; VF, Ventricular fibrillation.
P < 0.05 was considered statistically significant.

© 2024 by the authors. This article is an open access article distributed under the terms and conditions of the Creative Commons Attribution (CC BY 4.0) license.
